# Chemical and Molecular Strategies in Restoring Autophagic Flux in TDP-43 Proteinopathy

**DOI:** 10.3390/molecules31060924

**Published:** 2026-03-10

**Authors:** Angelo Jamerlan, John Hulme

**Affiliations:** Department of Bionanotechnology, Bionano Research Institute, Gachon University, Seongnam 13120, Gyeonggi-do, Republic of Korea; angelo@gachon.ac.kr

**Keywords:** TDP-43 proteinopathy, autophagy-lysosome pathway, proteostasis, neurodegeneration, autophagic flux, PROTACs, frontotemporal dementia, amyotrophic lateral sclerosis

## Abstract

The cytoplasmic accumulation of TDP-43 aggregates remains a persistent pathological hallmark of neurodegenerative diseases, including amyotrophic lateral sclerosis (ALS), frontotemporal dementia (FTD), and limbic-predominant age-related TDP-43 encephalopathy (LATE). The cell’s natural clearance mechanisms, the Ubiquitin-Proteasome System (UPS) and the autophagy-lysosome pathway (ALP), are hypothesized to fail, at least in part, due to the sequestration of key components of these pathways by pathological TDP-43 species, thereby impairing autophagosome-lysosome fusion and lysosomal competence. Classical autophagic activators (e.g., rapamycin) can initiate upstream steps in the pathway but cannot address downstream flux bottlenecks, limiting their ability to restore effective TDP-43 clearance. This review revisits classical strategies and discusses newer approaches to modulate TDP-43 clearance, including transcription factor EB (TFEB) activators, proteolysis-targeting chimeras (PROTACs), and antisense oligonucleotides (ASOs). We propose that adopting multi-targeting strategies and developing better biomarkers are vital for clinical success.

## 1. Introduction

The formation of protein aggregates within neurons is a typical clinical hallmark of neurodegenerative diseases [1,2,3,4]. Neurons are particularly vulnerable to this aberrant accumulation due to their unique biology, increased metabolic demand, and post-mitotic status. Due to their limited division, neurons are unable to dilute the aggregates, thus these cells are vulnerable to cumulative damage [5,6]. Particularly, the accumulation of trans-active response DNA-binding protein (43 kDa) (TDP-43) in amyotrophic lateral sclerosis (ALS), frontotemporal dementia (FTD), and recently, limbic predominant age-related TDP-43 encephalopathy (LATE; a form of dementia strikingly similar to Alzheimer’s disease (AD), but affects the limbic areas and is generally characterized by TDP-43 cytoplasmic aggregation) [7] has long intrigued experts and complicated the neurodegenerative disease landscape, underscoring its multifaceted nature [8,9,10].

TDP-43 is a ubiquitously expressed RNA-binding protein with diverse regulatory roles in RNA metabolism and gene expression. This protein primarily resides in the nucleus, packaged in ribonucleoprotein (RNP) granules, but constantly shuttles between the nucleus and cytoplasm to perform its functions [11,12]. It can directly bind to DNA (TG-repeats) to regulate gene expression [13], interact with heterogeneous nuclear ribonucleoproteins (hnRNPs) to enable splice site selection and regulate pre-mRNA splicing [12,14,15], and facilitate the nucleocytoplasmic shuttling of RNA [16]. TDP-43 can also regulate its own expression by binding its mRNA in a negative feedback loop, helping maintain its precise concentrations in the cell [17,18]. However, the multifaceted nature of TDP-43 also makes it particularly vulnerable to various insults that disrupt its normal functions. Mutations can increase TDP-43’s stability, prolonging its half-life, making it more difficult to be processed by the UPS, and forming more stable aggregates [12]. External stressors, such as oxidative stress, can also decrease protein solubility [19]. These factors increase the risk of forming irreversible aggregates that hinder TDP-43 from executing its critical regulatory functions, ultimately leading to cell death.

Approximately 97% of ALS cases and 45% of FTD (FTD-TDP) cases exhibited TDP-43 aggregation [20,21], and a significant secondary pathology was also observed in AD and LATE with neuropathological changes (LATE-NC) cases [22,23,24]. The consequences of the accumulation of these aggregates are still highly debated, with questions remaining about whether the toxicity is plaque-intrinsic (gain-of-function) or more akin to sinks that sequester and inactivate functional TDP-43, leading to dysregulation of essential cellular functions (loss-of-function). However, earlier studies have indicated that TDP-43 pathogenicity is potentially a combination of both mechanisms [25,26,27]. Nevertheless, one pathological explanation of this aberrant accumulation posits that it is primarily driven by the impairment of cellular systems that clear these aggregates: the ubiquitin-proteasome system (UPS) for misfolded soluble monomers, and the autophagy-lysosome pathway (ALP) for larger aggregates [28,29].

This clearance deficit hypothesis suggests that TDP-43 proteinopathy is driven at least as much by the breakdown of degradation systems as by production or intrinsic aggregation; thus, restoring defective proteostasis and clearance systems becomes a viable therapeutic target. The UPS primarily processes soluble, misfolded TDP-43 monomers in the nucleus, while the ALP processes the larger cytoplasmic aggregates. These two systems are restricted to their target protein species, meaning that the failure of one results in the accumulation of its target species [28]. Studies have demonstrated that autophagy disruption plays a significant role in the pathogenesis of ALS and FTD, and that aberrant TDP-43 could interfere with lysosomal fusion and function, further contributing to its own uncontrolled cytoplasmic deposition [30,31]. It is therefore logical that stimulating these pathways could enhance proteostatic mechanisms, thereby promoting the clearance of TDP-43 aggregates.

In this review, we will discuss the strategies based on this rationale as potential therapeutic avenues for TDP-43 pathologies. We build our discussion around a propagating “vicious cycle” model in which pathological TDP-43 species increasingly burden and could compromise the UPS and ALP, particularly through ubiquitin-dependent tagging, endolysosomal trafficking, and autophagosome-lysosome fusion, thus adding to proteostatic collapse and impaired autophagic flux. While previous work (studies discussed in detail in Section 4) mainly focused on the generic activation of autophagy, our review prioritizes restoring functional flux and implementing multi-target strategies as improved therapeutic interventions.

## 2. Regulation of TDP-43 Monomer Accumulation via UPS Activation

The pathological cascade leading to TDP-43 aggregation begins with increased accumulation of its monomeric form. Apart from its natural tendency to aggregate (which can be further enhanced by mutations) [10], TDP-43 in postmortem brains and spinal cords of patients with sporadic ALS was reported to be unable to form multimers or dimers [32]. Furthermore, N-terminal dimerization-deficient TDP-43 was found to comprise pathological inclusion bodies in ALS motor neurons [32]. Several factors, such as post-translational modifications (PTMs), such as acetylation, citrullination, C-terminal phosphorylation, contribute to the instability of RNA binding in the RNA recognition motifs (RRMs), exposing regions that cause the monomer to misfold and undergo liquid–liquid phase separation (LLPS) [33,34,35,36,37]. Misfolding of the monomers can bury lysines and linear degrons that E3 ligases and UPS shuttles recognize, while exposing other residues that favor self-association and prevent proteasome processing. This could kinetically divert the monomers toward phase separation rather than tagging them for UPS degradation [4,28,38].

### 2.1. The Role of Ubiquitination

Ubiquitination is an essential PTM that tags aberrant proteins for degradation by the UPS. Various ligases and factors drive this process (e.g., UBE2E3, parkin, VHL/CUL2, Znf129, etc.) [39]. However, this system appears vulnerable to increased TDP-43 aggregation, which has been reported to sequester UPS components and deplete the free ubiquitin pool, possibly blunting the efficient execution of the UPS cascade [39]. In other words, once TDP-43 oligomers begin to seed due to mutations and aberrant PTMs, they interact with and sequester essential UPS components (e.g., E2/E3 enzymes and ubiquitin), effectively disabling the very machinery that facilitates their own degradation. Therefore, fine-tuning the ubiquitination architecture rather than global depletion of aggregated TDP-43 was proposed as a viable therapeutic strategy to restore proteolytic flux and mitigate disease progression [39].

Early findings by Hans et al. on *Drosophila* models have identified ubiquitin-conjugating enzymes (UBE2E) and ubiquitin isopeptidase Y (UBPY) as modulators of TDP-43 ubiquitination. They recognized that the UBE2E class of enzymes promotes ubiquitination, while UBPY reduces it [40]. However, UBE2E overexpression failed to reduce TDP-43 steady-state levels within the observation window as one would predict for proteasome-targeting ubiquitination. Moreover, forced ubiquitination by UBE2E3 shifts TDP-43 into insoluble fractions, and the authors attributed this to modifications in TDP-43’s tertiary or quaternary structure [40]. However, RNAi knockdown of DUB UBPY in *Drosophila* led to the accumulation of insoluble ubiquitinated TDP-43, thereby enhancing neurotoxicity. Logically, activation of UBPY could reduce insoluble ubiquitinated TDP-43 species; however, this parameter was not explicitly tested in the study. Nevertheless, the authors concluded that UBPY is a disease-modifying factor that could potentially suppress TDP-43 neurotoxicity [40].

More recently, Byrd et al. employed an unbiased yeast genome-wide screen using high-throughput dot blots and identified ESCRT complex factors (which induce membrane invagination) and K63-linked ubiquitination as key facilitators of TDP-43 endolysosomal clearance [41]. Moreover, NEDD4, a HECT-type E3 ubiquitin ligase, was found to be involved in TDP-43 ubiquitination [41]. Additional transfection experiments that overexpressed NEDD4 rescued the reduction in cell viability that resulted from the overexpression of TDP-43-GFP and TDP-35-GFP [41]. Taken together, the findings suggest the therapeutic potential of NEDD4, although further studies are needed to confirm its clinical efficacy.

### 2.2. Other Strategies Involving the UPS

Several strategies have been developed to enhance proteasomal activity. In one early study, affinity probes based on pyrazolones, five-membered aromatic heterocyclic compounds [42], were synthesized and used to screen for high-affinity binding partners. This class of compounds was previously identified through a high-throughput screening of a >50,000-compound library in a G93A-SOD1 cell model [43]. Pyrazolones were confirmed to be neuroprotective in PC12-SOD1^G93A^ cells [44]. Using proteomics, the regulatory subunits of the proteasome: PSMC1, PSMC3, and TCP-1, were characterized as putative targets of the probes [44]. Subsequent proteasome activation by the pyrazolones was demonstrated in the absence of exogenous proteasome inhibitors, as well as through the restoration of degradative function (using a fluorogenic substrate) in the cell model [42], making the probes among the first candidates with a promising ability to enhance proteasomal function.

The expression of HDAC6, a cytosolic deacetylase enzyme essential in the regulation of protein quality control at the interface between UPS impairment and compensatory autophagy, was modulated using plasmid transfection (overexpression) and siRNA (knockdown) [45]. Here, the authors demonstrated that HDAC6 overexpression decreased insoluble and cytosolic TDP-43 levels in a TDP-43-overexpressing cell model. Conversely, knockdown of *Hdac6* increased total TDP-43 levels [45]. LC3-I/II levels (ubiquitin-like adaptor proteins on autophagosome membranes that facilitate phagophore expansion and protein recruitment) were also monitored and found to be significantly increased in TDP-43-overexpressing cells [45]. Their concentrations increased further when HDAC6 expression was enhanced, whereas *Hdac6* knockdown completely abolished LC3-I/II levels. These findings suggest that HDAC6 regulates TDP-43-induced UPS impairment through the ALP. The authors confirmed this hypothesis through Bafilomycin A1 (Baf), an autophagy inhibitor, and found no changes in TDP-43 levels despite HDAC6 overexpression [45].

Another study showed that the IκB kinase (IKK) complex, which typically phosphorylates IκB proteins for UPS degradation [46], could directly phosphorylate TDP-43 for UPS degradation [47]. IKKβ, together with IKKα and the scaffold subunit NEMO, is required for efficient phosphorylation of aberrant TDP-43 and its subsequent targeting to the UPS. Moreover, phosphorylation of the N-terminal residues Thr8 and Ser92 was identified as critical for the IKK-dependent reduction in cytoplasmic TDP-43 levels [47]. Most importantly, IKKβ was also shown to reduce only cytoplasmic aggregation-prone TDP-43 and had no considerable effect on wt-TDP-43 concentrations [47], suggesting that modulating IKKβ activity could represent a strategy for preferentially targeting pathological cytoplasmic TDP-43 while sparing physiological nuclear TDP-43.

Much more recently, the knockdown of RAD23A (via an inducible mutant TDP-43 HEK293 cell line), a gene coding for a member of the Rad23 family of DNA repair/ubiquitin-proteasome shuttle proteins, reduced insoluble TDP-43 levels in the cell model as well as primary rat cortical neurons expressing A315T mTDP-43 [48]. Using a proteomic screen, USP13, a deubiquitinase, was found to be related to this cascade and a modifier of TDP-43-induced aggregation and cytotoxicity [48]. Knockdown of this protein also reduced the sarkosyl-insoluble mTDP-43 in both cell models, and reduced cell death of the rat motor neurons and improved locomotor deficits in *C. elegans* ALS models, making RAD23A and USP13 possible therapeutic targets for TDP-43 clearance [48].

Taken together, several routes or components could be harnessed to enhance the efficiency of the UPS in serving as a critical defense against the accumulation of pathological TDP-43 monomers. Nevertheless, the recurring evidence of UPS component sequestration and the reliance on compensatory mechanisms, such as HDAC6, suggest that UPS alone may be inadequate when the load of accumulated monomers exceeds the critical substrate threshold or transitions into insoluble oligomers. This limitation highlights the need for a secondary line of defense capable of clearing larger aggregates. Therefore, the development of therapeutic strategies must extend beyond the UPS to the cell’s bulk degradation machinery: the ALP.

## 3. Molecular Mechanisms of TDP-43 Clearance via Autophagy

### 3.1. Macroautophagy Pathway

The autophagic system is a conserved intracellular system for degrading long-lived proteins and organelles in lysosomes [49,50,51]. Three main types of autophagy have been described in the current literature: macroautophagy, microautophagy, and chaperone-mediated autophagy (CMA) [50,51]. Among the three types described, macroautophagy and CMA were reported to be associated with the degradation of TDP-43 aggregates [52,53]. Macroautophagy involves the initial sequestration of the aggregates within autophagosomes, which, upon maturation, shuttle their cargo to fuse with the hydrolase-containing lysosome for proteolysis [51]. Interestingly, studies have shown the dual properties of TDP-43, where its presence regulates autophagy while also acting as the substrate of the process.

For instance, Leibiger et al. demonstrated that the endosomal vacuolar pathway and the vacuolar fusion machinery were critical for TDP-43 clearance and cell survival. In contrast, autophagy had a more complex and context-dependent contribution [31]. Interestingly, the study showed that TDP-43 interfered with lysosomal pathways and its own clearance [31]. Thus, it was suggested that in the absence of endolysosomal activity, autophagy facilitates TDP-43 clearance but simultaneously enhances TDP-43-triggered cell death, indicating that autophagy can be cytotoxic in this context despite its degradative role. These findings suggest that therapeutic strategies for TDP-43 proteinopathies should initially focus on restoring endolysosomal flux and vacuolar/lysosomal fusion, while carefully regulating autophagy to promote clearance, thereby avoiding lethal, TDP-43-dependent self-toxicity.

### 3.2. Chaperone-Mediated Autophagy (CMA)

CMA is a more selective process that operates via a cytosolic chaperone that recognizes a lysosomal targeting motif [51,54] and shuttles proteins to the lysosomal surface. The substrate proteins then interact with a membrane receptor, where lysosomal chaperones facilitate their entry into the lumen [51]. The selectivity of this pathway is beneficial under conditions that require minimal oxidative stress-induced damage or exposure to various toxic compounds, as only damaged proteins are removed without affecting intact ones [51,55,56]. In a study by Ormeño et al., the CMA was shown to be involved in the degradation of recombinant TDP-43 (rTDP-43). The authors demonstrated that CMA-positive lysosomes in rat liver specifically degraded rTDP-43 and contained endogenous TDP-43, providing evidence that TDP-43 could be a CMA substrate in vivo [53]. Previous studies have shown that CMA degradation occurs through the interaction of a specific targeting sequence (KFERQ) within the target protein with the heat shock cognate 70 kDa (Hsc70) chaperone [54,57,58,59]. The formed complex then interacts with the lysosomal-associated membrane protein isoform 2A (Lamp2A), which subsequently facilitates the translocation of the target protein into the lysosomal lumen [53]. The authors further investigated the compatibility of the CMA pathway for TDP-43 degradation through a competition assay using glyceraldehyde 3-phosphate dehydrogenase (GAPDH), an established CMA substrate [60]. A constant concentration of rTDP-43 was incubated with increasing concentrations of GAPDH, revealing complete TDP-43 degradation in the absence of GAPDH. In contrast, no TDP-43 degradation was observed in samples pre-incubated with GAPDH [53]. Moreover, an upregulation in CMA components (Hsc70 and Lamp2A) was observed in response to the overexpression of the aggregation-prone form of TDP-43. GAPDH concentrations were also compared in TDP-43-aggregate expressing cells at 24 and 72 h to check the possible influence of TDP-43 aggregates on CMA activity. Reduced concentrations were observed in the aggregate-expressing cells, with the decrease more evident at 24 h [53]. These findings indicate that the CMA pathway is also involved in TDP-43 degradation.

### 3.3. TFEB-Mediated Lysosomal Biogenesis

A parallel study by Xia et al. (2016) explored the consequences of TDP-43 nuclear loss by knocking down TDP-43 expression in HeLa (non-neuronal) and SH-SY5Y (neuronal) cells [61]. TDP-43 knockdown led to the nuclear translocation of transcription factor EB (TFEB), which is the master regulator of the ALP through the expression of autophagic gene products (e.g., ATG5, Beclin-1, and ATG9B) and lysosomal gene products (e.g., LAMP1, cathepsins, and subunits of ATPases) [61,62,63]. The translocation of TFEB was associated with the loss of raptor mRNA, leading to the disruption of mTORC1 activity and, consequently, a reduction in the efficiency of TFEB phosphorylation [61]. Since phosphorylation is required for TFEB to be sequestered in the cytosol, it translocates to the nucleus. It accumulates there, resulting in enhanced global gene expression involved in ALP and increased biogenesis of autophagosomes and lysosomes [61]. However, TDP-43 loss also impaired autophagosome-lysosomal fusion by reducing dynactin 1 expression, leading to the accumulation of immature autophagic vesicles that could not be processed [61]. This presents a scenario in which the significant upregulation of ALP components from an overactive TFEB (due to chronic mTORC1 inhibition) further drives the formation of autophagosomes that have nowhere to go, as they are unable to fuse with the lysosome due to a lack of dynactin 1, thereby overwhelming the ALP [61]. Therefore, restoring mTORC1 activity and physiological levels of dynactin 1 are potential strategies to mitigate the ALP’s failure to clear TDP-43 aggregates.

**Figure 1:**
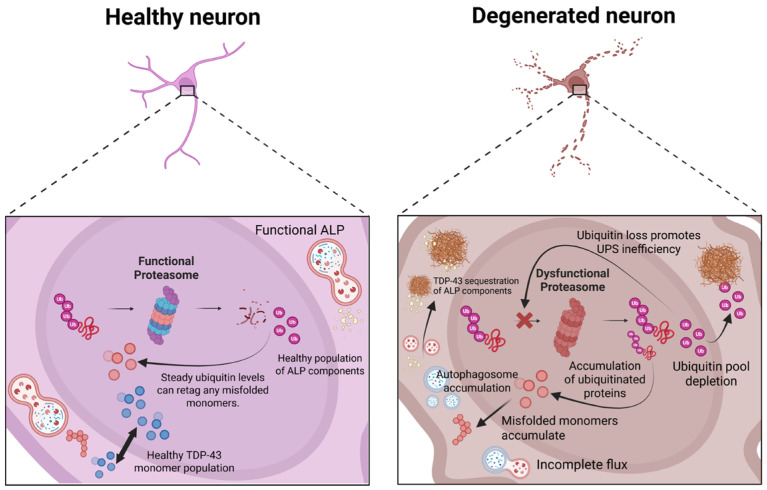
The vicious cycle of TDP-43-mediated proteostatic collapse. TDP-43 aggregates actively contribute to pathology rather than merely serving as passive metabolic waste. They sequester essential components of the UPS, such as ubiquitin and E2/E3 ligases, and disrupt the ALP by depleting related components (e.g., dynactin-1), which are essential for autophagosome-lysosomal fusion. This leads to a self-perpetuating “vicious cycle” in which TDP-43 disrupts the very complexes responsible for its clearance, thereby impairing autophagic flux. Created in BioRender. Angelo Jamerlan. (2025) https://BioRender.com/.

Collectively, the experimental data illustrate a model in which the UPS primarily processes soluble misfolded TDP-43, while oligomer and late-stage aggregates increasingly depend on endolysosomal and autophagic routes for clearance. Excess TDP-43 has been reported to sequester ubiquitin and UPS factors, alter TFEB-dependent lysosomal and autophagosomal biogenesis, and impair autophagosome-lysosomal fusion, resulting from reduced dynactin-1 and related trafficking defects, particularly in cellular and animal models. These changes could increasingly reduce clearance capacity, furthering TDP-43 accumulation and resulting in a self-reinforcing “vicious cycle” of proteostatic failure; the observed ubiquitin-positive TDP-43 inclusions and disturbed autophagic markers in human ALS/FTD tissue are consistent with this framework, but several mechanistic steps are still inferential and remain to be validated in patients. Figure 1 summarizes the vicious cycle that results from the accumulation of these aggregates.

## 4. Pharmacological Autophagy Enhancers: Preclinical Evidence

**Figure 2:**
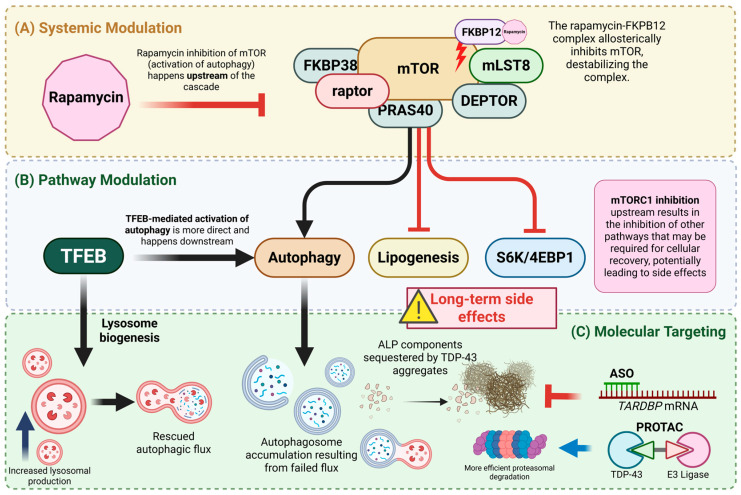
Nested metabolic hierarchy of therapeutic interventions of TDP-43 proteinopathy. The schematic illustrates the transition from broad metabolic regulation to more precise targeting. (**A**) Systemic modulation (**top panel**) involves upstream inhibition of mTORC1 by rapamycin, which activates autophagy but also exerts pleiotropic effects on lipogenesis and metabolism. (**B**) TFEB-mediated pathway modulation (**middle panel**) is more downstream, focusing on lysosomal biogenesis and restoring ALP fusion to bypass the bottlenecks created by TDP-43 sequestration. (**C**) Molecular targeting (**bottom panel**) uses precision tools such as antisense oligonucleotides (ASOs) and proteolysis-targeting chimeras (PROTACs), which provide the highest specificity by targeting TARDBP mRNA or TDP-43 directly, thereby preventing the initiation of the vicious cycle. Created in BioRender. Angelo Jamerlan. (2025) https://BioRender.com/.

Current and emerging interventions for driving the degradation of aberrant TDP-43 can be categorized by their specificity and their point of attack within the proteostatic network. These range from broad-spectrum mTOR-dependent inducers to newer high-precision molecular tools that target the transcript or the protein directly (Figure 2).

### 4.1. Systemic m-TOR-Dependent and Metabolic Modulators

The mTORC1 (mechanistic target of rapamycin complex 1) is a principal suppressor of autophagy that phosphorylates the main initiator of the cascade, the ULK-ATG13-FIP200 complex [64]. The inactivated complex prevents the formation of autophagosomes and the lysosomal recycling of cytoplasmic components. However, under cellular stress and pathological conditions, the mTORC1 is deactivated, and several components have previously been demonstrated to utilize the same pathway to enhance autophagic clearance of pathological aggregates. Table 1 summarizes these compounds along with others that engage other pathways to achieve the same effect. These compounds were also tiered according to their pre-clinical evidence, with tier 3 representing human trials, tier 2 animal models, and tier 1 in vitro models.

Rapamycin is a well-documented autophagy enhancer that showed promising results in clearing mutant huntingtin, α-synuclein, β-amyloid, and prions [65,66,67,68]. Treating TDP-43 transgenic mice (FTLD-U; frontotemporal dementia with ubiquitin-positive inclusions) rescued learning and memory, improved motor neuron function, and delayed pathological progression [69]. Tamoxifen treatment showed results similar to those observed with lowered lysosomal substrates and increased lysosomal markers [69]. Despite these promising results, the limitations of mTOR inhibition were revealed by the failure to rescue an ALS mouse model (mutant SOD^G93A^) phenotypically [70]. The same was true for other AD models [71,72,73], thus demonstrating that this approach is not generally applicable to all cases of neurodegenerative disease.

Interestingly, monepantel, a commonly used veterinary anthelmintic in livestock, demonstrates off-target inhibition in mTOR signaling [74,75]. In fact, an early clinical trial (Phase 1 MEND trial) demonstrated that monepantel treatment showed 40–60% improvement in functional decline, but should be interpreted cautiously pending larger, randomized trials [74]. Overall, the initial findings suggested good BBB permeability marked by monepantel sulfone in the CSF of MS patients, as well as good tolerance within small cohorts, but long-term human safety data are still limited [74].

Other pharmacological agents are considered mTOR-independent but have broad metabolic effects similar to those of their mTOR-dependent cousins. Spermidine, a polyamine in citrus and soybean, activates autophagy through SIRT1-mediated deacetylation [76]. Carbamazepine, an anticonvulsant and mood-stabilizing drug, activates autophagy through the depletion of myoinositol levels in the phosphoinositol pathway [77]. However, these results were limited to animal models, and carbamazepine lacks TDP-43-specific efficacy data (Table 1).

Metformin is another related drug that suppresses mTOR signaling through the indirect activation of AMP-activated protein kinase (AMPK) [78,79,80]. The metformin treatment of APP/PS1 mice decreased Aβ pathology [81]. Observational studies on diabetic patients likewise showed an associated lower risk of AD [82,83]. However, these findings remain controversial since other studies did not find an associated lowered AD risk in other cohorts [84], while others surprisingly showed an increased risk [85,86]. Thus, metformin seems better positioned as a background adjuvant rather than a primary TDP-43-clearing drug, given its long safety record and low toxicity, which could improve outcomes in future combination regimens.

Other natural compounds and polyphenols offer the same effects and will be discussed in detail in another section. These are also primarily systemic, mTOR-independent autophagic enhancers. Since these compounds target mTOR and broader pathways, the risk of off-target effects is much higher and could hinder cellular recovery.

### 4.2. Pathway-Selective TFEB, Autophagy, Kinase, and PDE Modulators

Unlike rapamycin and its rapalogs, mTOR-independent inducers trigger autophagy without measurably suppressing mTOR activity, often preserving anabolic signaling while still enhancing autophagic clearance. This is particularly important in chronic neurodegenerative settings, where long-term mTORC1 inhibition may lead to undesired off-target effects, making these pathway-selective strategies a more favorable approach.

Trehalose, a prominent non-reducing disaccharide, was shown to prevent in vitro aggregation and reduce the cytotoxicity of Aβ40/42 [82] and to inhibit the aggregation of pathologic huntingtin in a mouse model [87]. It was hypothesized that trehalose directly interacted with the pathologic proteins to disrupt their aggregation [87]. Wang et al. (2018) tested the inhibitory effects of trehalose in a cell culture model and, through measurable increases in LC3-I and LC3-II conversion and immunofluorescence, demonstrated autophagy activation via the TFEB pathway [88]. However, increased LC3-II levels alone are only indicative of autophagosome formation and do not necessarily reflect functional flux. Subsequent studies revealed that trehalose treatment increased autophagosome levels but did not affect functional autophagic flux [89]. It was also shown that trehalose treatment resulted in the inefficient delivery of LC3 from autophagosomes to autolysosomes [90]. Therefore, it may be essential to examine the restoration of autophagic flux as a parallel observation to increased autophagosome levels to increase the certainty of ALP restoration in succeeding studies. As summarized in Table 2, LC3-II accumulation should thus be interpreted in the context of cargo turnover, tandem LC3 reporters, and fusion/lysosomal competence assays to differentiate actual flux restoration from simple autophagosome biogenesis.

Trehalose activation of PPP3CB (calcineurin), a phosphatase that removes the inhibitory phosphate groups of TFEB, was also suggested to be involved in TFEB-mediated autophagy [91,92]. Despite these promising results, the bioavailability of trehalose is severely limited by intestinal TREH (trehalase [brush-border membrane glycoprotein]) when the compound is taken orally [93], requiring intravenous administration. Nevertheless, TREH-resistant analogs, such as melibiose and lactulose, have been shown to promote TFEB nuclear translocation and may be considered potential TFEB modulators with improved bioavailability [91].

Ibudilast is another candidate that inhibits phosphodiesterases (PDEs; e.g., cAMP, cGMP), which are essential secondary messengers in muscle tone regulation, inflammatory cell activation, and epithelial barrier function. Clinical trials for the treatment of multiple sclerosis (MS, ongoing Phase III) and ALS (active Phase IIb) showed neuroprotective effects in progressive MS [94], while ALS data suggest mechanistic compatibility but remain inconclusive pending full trial results [95].

Chen et al. (2020) showed that ibudilast treatment enhanced the clearance of TDP-43 and SOD-1 aggregates in HEK-293 cells [96]. The authors demonstrated that, despite the drug being primarily a PDE inhibitor, it could also enhance autophagy through the TFEB pathway and the inhibition of mTORC1 [96]. This data suggests that although the drug is not an mTOR-dependent autophagic enhancer in the classical sense, since it modulates signaling molecules upstream of mTORC1, decreased mTORC1 activity is somehow still required to accomplish full aggregate clearance via autophagy [96].

Several other TFEB activators have been investigated, including trametinib, clomiphene citrate, and levacetylleucine (NALL), which demonstrated reduced Aβ accumulation, phenotypic rescue, and maintenance of lysosomal integrity [97,98,99]. Interestingly, Davis et al. (2025) showed that the D-enantiomer of the NALL backbone could antagonize the effects of its L-enantiomer, despite the N-acetyl moiety only being involved in TFEB activation [99]. This suggested that stereochemistry may also significantly affect pharmaceutical efficacy and should be investigated further.

Recently, multiparameter high-throughput screening differentiated kinase inhibitors that activated TFEB from those that activated lysosomal function. This was done by evaluating the nuclear translocation of TFEB and TFE3, a related transcription factor that also drives the expression of autophagy-lysosome and stress-response genes [100]. Using the Published Kinase Inhibitor Set 2 (PKIS2) library, 74 hits were found and included AKT-targeting 4-aryl-7-azaindoles that enhanced lysosomal activity and some 2-aryl-4-anilino(pyridine-4-yl)-quinazoline series (e.g., NK140, NK176, NK177) that activated TFEB and TFE3, upregulated CLEAR genes, and drove the clearance of mutant HTT aggregates [100]. This implied that TFEB/TFE3 activation and CLEAR gene expression could be achieved without the lysosomal dysfunction typically observed in chloroquine and related lysosomotropic compounds. However, the same compounds have not yet been tested for TDP-43 clearance.

Kinase inhibitors that enhance autophagy typically modulate upstream or parallel signaling nodes (e.g., ERK, PDGFR-Akt, AMPK) and exert their effect on the ULK complex only secondarily, resulting in the added preservation of mTOR’s anabolic functions while increasing autophagic flux or lysosomal biogenesis. Bosutinib, a drug for treating chronic myelogenous leukemia (CML), was found using a phenotypic drug screen (using iPSC-derived motor neurons expressing the SOD mutation) to target the Src/c-Abl signaling axis, which controls cell survival, cytoskeletal dynamics, and the stress response [101]. Autophagy was enhanced, misfolded SOD1 protein levels were reduced, and the survival of neurons with mutant TDP-43 and C9orf72 expansions was increased. ALS clinical trials initially showed the drug to be safe and well-tolerated, and the reported adverse effects were typical of CML and unrelated to ALS [102]. A Phase II observational study in Japan is ongoing and was designed as a real-world, observational effectiveness study using routinely collected clinical data for evaluation [103,104].

## 5. Polyphenols and Other Naturally Derived Molecules

Naturally derived compounds and polyphenols are being recognized as viable candidates for enhancing TDP-43 clearance. They have been shown to influence aggregation and toxicity while promoting neuronal resilience and to exhibit milder toxicity profiles than other synthetic molecules [105]. In contrast to more target-specific synthetic small molecules, natural compounds are typically characterized as multitarget, pleiotropic modulators of proteostasis and autophagy, often acting simultaneously at multiple nodes (e.g., redox balance, inflammatory signaling, mitochondrial function, and nutrient-sensing pathways) [106]. Their pro-autophagic effects typically result from indirect modulation of multiple pathways rather than direct binding to a single autophagy protein. Since multiple stress pathways contribute to TDP-43 pathology in chronic diseases, a therapeutic strategy that simultaneously enhances autophagy and provides antioxidant, anti-inflammatory, and mitochondrial protection may offer synergistic advantages [106,107]. These compounds also exploit overlapping mechanisms with synthetic drugs, but do so through upstream signaling rather than by directly inhibiting mTORC1 or kinases. This multi-pathway engagement may produce wider cytoprotective effects, but could also result in a more complex pharmacological profile and ambiguous effective central nervous system (CNS) exposures [108,109,110]. This section focuses on polyphenolic natural products such as curcumin, epigallocatechin-3-gallate (EGCG), and resveratrol, as well as other naturally derived small molecules, including withaferin A, that modulate autophagy and TDP-43 pathology.

### 5.1. Curcumin

Curcumin is a natural compound derived from the powdered rhizome of *Curcuma longa*, more commonly known as turmeric [107,111]. It is a popular and widely studied polyphenol due to its broad-ranging benefits, including antioxidant, anti-inflammatory, antiproliferative, antitumor, analgesic, and anti-amyloid properties [107]. Some studies further suggest its safety as a bioactive compound, even when administered in high doses [112], which adds to its broad appeal. In terms of autophagy activation, curcumin has been documented to stimulate the PI3K/Akt/mTOR, AMPK, MAPK/ERK1/2, Bcl-2, and Rab GTPase pathways [113].

Several studies have investigated the therapeutic potential of different curcuminoids in the treatment of ALS. One demonstrated the capability of bisdemethoxycurcumin (BDC) and its analogs to clear misfolded/aggregated Aβ/SOD1 in innate immune cells (U-937) by potentiating the expression of MGAT3, VDR, and TLR [114]. A distinct subset of anti-inflammatory curcuminoids was also described that potently inhibited COX-2 and leukotriene B4, as well as IL-17A, IL-6, IL-10, and TNF-α in SOD1-stimulated peripheral blood mononuclear cells from patients with sporadic ALS [114]. However, the study did not disclose the categorization of individual curcuminoids in each set; thus, potential overlap between SOD1/Aβ1-clearance-enhancing and anti-inflammatory analogs cannot be determined from the published data [114]. A later study by Song et al. (2016) revealed a new curcumin analog (C1) that activates TFEB by binding to its N-terminus, promoting its nuclear translocation without inhibiting MTOR [115]. As a result, both autophagy and lysosomal pathways are enhanced in vitro and in vivo [115]. This study also confirmed increased autophagy flux by measuring the degradation of sequestosome 1 (SQSTM1), a selective autophagy substrate. The cells were treated with cycloheximide to prevent unintended upregulation of SQSTM1 caused by the stimulated TFEB [115]. Recently, solid lipid curcumin particles (SLCP) were administered orally to female Prp-TDP-43^A315T^ mice, resulting in a significant reduction in pathological insoluble phosphorylated TDP-43 species, attenuation of disease progression, and improved survival and weight loss by regulating estradiol levels through CYP19A1 upregulation and CYP3A4 downregulation [116]. This implicated a CYP450-estrogen pathway rather than autophagy activation as the primary mechanism of action.

Curcumin is typically known as an autophagy modulator, but its influence on autophagy flux is highly context-dependent [107,109]. Many neurodegenerative and injury models (AD cells, SCI, aging cardiomyocytes) demonstrated that curcumin promotes autophagic flux or at least induces autophagy, usually through the inhibition of PI3K/Akt/mTOR or activation of AMPK/SIRT1 [117,118,119]. This correlates with the improved clearance of toxic proteins or damaged organelles; thus, autophagy is inferred to be protective in these scenarios [117,118,120]. In contrast, curcumin acts as a neuroprotective agent by inhibiting excessive or abnormal autophagy under severe, sustained stress conditions, such as H_2_O_2_-induced oxidative stress in neural stem or progenitor cells or ischemia–reperfusion models [118,121]. This regulation helps to normalize LC3-II/LC3-I, Beclin1, p-ERK, and p62 expression, thereby restoring proper autophagic flux [122,123]. Thus, autophagy-associated cell death is prevented, rather than driving further degradation. Considering these findings together, it seems that curcumin acts as an autophagic “buffer,” favoring autophagy when aggregate clearance is insufficient (e.g., increased accumulation, impaired axonal transport), but begins to dampen its effects when autophagic overstimulation contributes to increased cell death or maladaptive responses [117,118,123]. This likely reflects curcumin’s action at multiple upstream nodes (PI3K/Akt/mTOR, AMPK, SIRT1, ERK, ROS/p62-Keap-Nrf2), so the overall effect will depend on the cell’s current signaling state and the nature of the insult [107,122].

An early study by Duan et al. (2014) showed in mutant TDP-43^Q331K^ and TDP-25 neuronal cells (NSC-34) that treatment with various curcumin derivatives reduced levels of TDP-43 fragments [124]. Notably, monocarbonyl dimethoxycurcumin C (Compound C) significantly reduced the expression levels and aggregates of TDP-25-transfected cells [124]. Reduced lactate dehydrogenase (LDH) and malondialdehyde (MDA) levels in the NSC-34, following Compound C treatment, demonstrated the compound’s role in reducing oxidative stress [124]. The authors also showed that the compound significantly induced heme oxygenase-1 (HO-1), an antioxidant enzyme, compared with the other candidates. They previously showed a correlation between reduced HO-1 expression and mt-TDP-43 expression, which could contribute to increased oxidative damage [125]. Further investigation suggested that Compound C-induced HO-1 was only partially involved in TDP-43 fragment degradation [124].

Despite its biological potential, curcumin’s therapeutic applicability is severely limited by poor bioavailability, rapid metabolism, and low BBB permeability [126,127,128]. Novel delivery systems, such as solid lipid particles, are being explored to overcome these hurdles [129,130].

### 5.2. EGCG

Epigallocatechin-3-gallate (EGCG), a polyphenol derived from green tea, has been reported to bind to TDP-43 with micromolar affinity and significantly prevent nucleation, thereby hindering TDP-43 aggregation in vitro and redirecting the protein to form less toxic oligomeric species [131,132]. EGCG is also a characterized modulator of autophagy in other cell types through AMPK/mTOR/ULK1 and CaMKKβ-dependent pathways, but a direct association of EGCG in the promotion of autophagic clearance of TDP-43 inclusions has not been reported, and current evidence mainly supports an anti-aggregation mechanism rather than cargo-specific engagement of the ALP [133,134].

### 5.3. Resveratrol

Resveratrol (RSV) is a plant-derived polyphenol found in fruits and nuts that acts as a defense compound against environmental stress and pathogens [135,136]. Its antioxidative and anti-inflammatory properties allow it to scavenge reactive oxygen species (ROS) and influence redox-sensitive pathways [137]. Its suggested benefits (e.g., cardioprotective, neuroprotective, antitumor, anti-aging, and other metabolic effects) have made it widely available as a dietary supplement [136]. Earlier studies have demonstrated the SIRT-1-activating role of RSV, with an important nuance that could lead to assay artifacts: RSV can induce conformational changes in SIRT-1, making it preferentially bind fluorophore-labeled substrates that do not necessarily resemble its physiological targets [138]. Nevertheless, a subsequent study showed that RSV treatment reversed decreased SIRT1 and FOXO3a expression in human monocytic cells (THP-1) under hyperglycemic conditions [139]. Building on the knowledge that SIRT-1 influences the nuclear translocation of TFEB, resulting in the activation of the autophagy-lysosomal pathway, a study by Bao et al. (2016) used RSV as a SIRT-1 activator to stimulate microglial expression of TFEB in BV2 murine microglia [140]. Here, they demonstrated that the upregulated TFEB facilitated the degradation of fibrillar Aβ and reduced plaque formation by increasing lysosomal biogenesis and activity [140]. Crucially, they also revealed a key mechanistic detail: SIRT-1 activates TFEB by deacetylating it at Lys-116 [140]. More recently, another study uncovered a TFEB-related pathway, which is an endoplasmic reticulum (ER)-Ca^2+^ signaling cascade, as well as another cofactor, protein phosphatase 2A (PP2A), which dephosphorylates TFEB [135]. Based on these findings, RSV appears to be a TFEB activator that integrates SIRT-1, ER-Ca^2+^ signaling, and PP2A-mediated dephosphorylation. However, whether such TFEB activation is enough to drive TDP-43 clearance in neurodegenerative disease models remains unclear and requires further investigation.

### 5.4. Withaferin-A and Analogs

Withaferin-A (WFA) is an active withanolide derived from the medicinal herb *Withania somnifera*, and was shown to induce autophagy, reduce TDP-43 proteinopathy (RIPA-insoluble fraction), and improve cognitive function in transgenic FTLD mice expressing mutant TDP-43^G348C^ [141]. WFA also demonstrated anti-inflammatory effects by reducing NF-κB activity and neuroinflammation in the mouse brain, and by increasing LC3BII [141]. However, the compound failed to modulate other autophagic markers, including Beclin-1, p62, and Atg-5 [141]. The reason remains unclear and contradicts another study, which found increased LC3BII, Atg-5, and Beclin-1 expression when the IκB-super-repressor transgene—mutant IκBα that blocks canonical NF-κB activation—was expressed in TDP-43^G348C^ mice [142]. Further investigation is required to resolve these inconsistencies, particularly given that LC3BII expression reflects only increased autophagosome formation, which could stem from impaired degradation rather than enhanced autophagy. To accurately distinguish between enhanced autophagic flux and simple accumulation of autophagosomes in the above results, flux assays (e.g., lysosomal inhibition with chloroquine/bafilomycin, LC3 turnover assays, or LC3 immunostaining to assess autolysosomes) may be required in future studies employing WFA.

Another study explored the effects of IMS-088, a novel WFA analog and an antagonist of nuclear factor-κB essential modulator (NEMO), on vascular dementia [143]. The authors examined chronic cerebral hypoperfusion (CCH), which refers to a long-term reduction in blood flow to the brain caused by vascular disorders linked to conditions such as hypertension, diabetes, and atherosclerosis [144]. CCH is recognized as a major contributor to vascular dementia in older adults. It was induced experimentally in mice through unilateral occlusion of the common carotid artery, leading to chronic cerebral hypoxia and metabolic stress without causing immediate extensive infarction. This led to cytoplasmic mislocalization of TDP-43 in neurons, insoluble phospho-TDP-43 aggregates, chronic microglial activation, and the development of cognitive deficits and motor impairments [143]. Orally administering IMS-088 in CCH mice mitigated TDP-43 pathology, increased autophagy, and improved mental and motor deficits [143]. Moreover, LC3B1 and 2, as well as Beclin-1 levels, were upregulated following IMS-088 treatment, indicating increased autophagy, which likely promoted the clearance of phospho-TDP-43 aggregates [143].

## 6. Advanced Therapeutic Modalities

The results of mitigating TDP-43 aggregation and other neurodegenerative proteins through the modulation of clearance pathways are promising; however, relying solely on these direct approaches is insufficient to address the complexities of proteinopathies. Classical mTOR-dependent autophagy activators, such as rapamycin and related agents, have been shown to reduce inclusions and improve behavioral deficits in mouse models. However, they act broadly on multiple targets, have intrinsic toxicities, and could be dampened in settings where TDP-43-associated lysosomal and autophagic flux deficiencies become dominant [31,49,61,69].

To date, there are no disease-modifying therapies that directly correct TDP-43 mislocalization, aggregation, or loss of nuclear function in neurodegenerative diseases, highlighting both the incomplete control of TDP-43 clearance and the fact that enhancing proteostasis does not eliminate toxic species without unwanted effects [31,145]. Since TDP-43 can also interfere with the very systems that clear it up, classical approaches are vulnerable to self-amplifying loops of proteostatic failure; hence, the need for precision tools that directly recognize TDP-43 species for removal or restoration of specific functions.

### 6.1. Proteolysis-Targeting Chimeras (PROTACs)

PROTACs have been gaining attention due to their demonstrated potential to target proteins associated with neurodegenerative diseases, specifically [146,147]. PROTACs are heterobifunctional molecules that typically consist of an E3-ligase-recruiting moiety and a ligand for the targeted protein, linked by a linker. Upon forming a ternary complex, the molecular scaffold induces proximity that facilitates the transfer of ubiquitin to the target protein through the UPS [148]. Since these molecular scaffolds can be “reused” in a sense, they do not require the full stoichiometric concentrations required in traditional compounds [149]. A small amount of PROTAC is sufficient to tag and eliminate all levels of the target protein, thereby reducing the risk of side effects associated with larger doses [150].

Early proof-of-concept studies by Buckley et al. (2015) utilized HaloTag fusion proteins to demonstrate that small-molecule PROTACs could successfully recruit VHL E3 ligase to degrade specific targets [151]. While this validates the mechanism, the requirement for foreign fusion tags limits clinical application, primarily when vulnerable areas, such as the CNS, are targeted, raising concerns about safety and immunogenicity.

In a more recent study, Tseng et al. (2023) designed and characterized four kinds of PROTACs with varying PEG linker lengths (2–5 ethylene glycol units) and showed via the filter trap assay that PROTAC-2 was the only candidate that facilitated the significant degradation of C-terminal TDP-43 aggregates (C-TDP-43) and alleviated C-TDP-43-induced cytotoxicity in Neuro-2a cells without impacting endogenous TDP-43 [152]. The C-TDP-43-binding end was a benzothiazole-aniline (BTA) derivative modified with a 6-O-PEG chain that connected to the linker. In contrast, the E3-ligase-recruiting end was a pomalidomide-based cereblon (CRBN) ligand, which was the same in all other PROTAC constructs [152]. PROTAC-2, with a linker length of 3 ethylene glycol units, was reported as the optimal candidate due to the most favorable spatial positioning between the BTA C-TDP-43 binder and CRBN recruiter, resulting in a more efficient and selective UPS-mediated degradation of toxic proteins [152].

Nevertheless, several challenges remain, including poor permeability through the BBB and target specificity. This is due to several factors, including the possible addition of new protein interfaces in the ternary complex that are absent in the binary complex, E3 ligase promiscuity, and dependence on structural epitope recognition rather than on the specific mutation [153,154,155]. Moreover, although abundant, E3 ligases in the brain exhibit limited diversity. More studies are needed to characterize their chemical properties, dynamic regulation, target specificity, and their specific role in the pathophysiology of neurodegeneration [156].

### 6.2. Antisense Oligonucleotides (ASOs)

The ASO-mediated modulation of transcripts associated with TDP-43 turnover has been explored in a limited but growing number of studies, suggesting a viable approach to regulating their levels and pathological accumulation. A prior study by Mann et al. (2019) featured an optogenetic expression construct that enabled selective induction of TDP-43 proteinopathy in HEK293 cells [157]. The observed TDP-43 aggregates resulted from the aberrant interactions within the low-complexity domains (LCD) of the proteins [157]. Here, the addition of ASOs acted as molecular “scaffolds” that helped stabilize the LCD through steric or allosteric restraints, thereby preventing pathological phase transitions [157]. These findings underscore the importance of maintaining the RRM domains of TDP-43 bound to RNA to preserve its molecular stability and reduce the likelihood of oligomerization and aggregation.

In a subsequent study, the application of ASOs was extended to the fused in sarcoma (FUS) protein, an RNA-binding protein related to TDP-43 that is also predominantly nuclear and modulates RNA metabolism under physiological conditions [158,159]. Here, a series of FUS knock-in mouse lines expressing FUS mutations found in ALS (FUS^P525L^ and FUS^ΔEX14^), which presented progressive, age-dependent motor neuropathy. A non-allele-specific FUS ASO (ION363) was demonstrated to efficiently silence Fus and reduce postnatal FUS levels in the brain and spinal cord [159]. The authors also showed that repeated intrathecal injections of ION363 in an ALS patient with FUS^P525L^ mutation lowered both wild-type and mutant FUS levels and markedly reduced FUS aggregates [159].

More recently, gapmer-type ASOs targeting TDP-43 have been developed using 2′-O,4′-C-ethylene nucleic acids (ENAs), which are more stable than traditional nucleic acids. Using a mouse model of ALS/FTD that expressed mutant human TDP-43, the authors demonstrated that the intracerebrovascular delivery of ENA-modified ASOs significantly reduced TDP-43 expression without any toxic consequences [160]. Notably, a single injection of ENA-modified ASOs resulted in the sustained improvement of behavioral abnormalities (e.g., anxiety-like behavior, hyperactivity) and the inhibition of cytoplasmic TDP-43 aggregation despite the restoration of TDP-43 initial levels [160].

Since TDP-43 dysfunction has multiple downstream consequences and the pathology itself is complex, targeting multiple downstream pathways could lead to better outcomes. A dual-modality strategy is being developed that can modulate faulty genes that influence the severity or progression of TDP-43 pathology and potentially correct specific TDP-43 loss-of-function phenotypes [161]. This approach could represent a significant shift in the field, given emerging mechanistic evidence that TDP-43 influences its own pathology by failing to modulate essential proteins, such as Stathmin-2 (STMN2), a microtubule-associated protein specifically expressed in neurons. This structural protein is required for axon outgrowth and maintenance, as well as for axonal regeneration following injury in vitro [162]. STMN2 loss in mice was reported to result in ALS-relevant pathology characterized by progressive neuropathy and significant loss of neuromuscular junctions [163]. Baughn et al. (2023) demonstrated that TDP-43 acts as a “steric blocker” that prevents the inclusion of a cryptic exon 2a into STMN2 pre-mRNA, which otherwise (due to loss of nuclear TDP-43) introduces an in-frame stop-codon and a premature polyadenylation signal that truncates the mature STMN2 mRNA, resulting in the loss of functional STMN2 [164]. The authors then developed ASO constructs that bind to exon 2a to prevent its inclusion, thereby rescuing regular STMN2 expression [164].

In developing ASOs for therapeutic targeting, emerging evidence has highlighted the need for alignment with specific repeat RNA species that most directly disrupt TDP-43 function, rather than simply reducing the number of pathological repeats. Notably, Rothstein et al. (2023) showed that ASOs targeting the antisense G_2_C_4_ repeat RNA of C9orf72 mitigated deficits in TDP-43 function in C9orf72 ALS/FTD patient-derived induced pluripotent stem cell (iPSC)- derived neurons (IPSNs) [165]. Previous clinical trials utilizing ASOs targeting the sense G4C2 repeat in C9orf72 ALS patients were terminated due to a lack of clinical efficacy [166,167]. The results in the Rothstein study showed that knocking down G_4_C_2_ does not correct the downstream consequences of C9orf72 ALS neurons, despite earlier data demonstrating a robust reduction in sense dipeptide repeats (DPRs), whereas G_2_C_4_ knockdown (in a 15 and 20-day period) restored regular expression and splicing of TDP-43 mRNA targets [165]. They have also demonstrated that G_2_C_4_ antisense RNA alone is sufficient to induce TDP-43 loss-of-function, leading to altered expression of numerous proteins that contribute to disease pathology [165]. These findings underscore that depending solely on histological examination (i.e., decreased RNA foci, aggregates) is not enough to infer the restoration of downstream TDP-43 function, as G_4_C_2_-sense-targeting ASOs could reduce repeat RNA/DPR pathology and improve neuronal survival, but fail to normalize TDP-43-controlled gene expression and cryptic exon inclusion in C9orf72 iPSNs.

### 6.3. Gene Therapy Approaches

As for gene therapy approaches targeting TDP-43 accumulation or enhancing its clearance, recent studies have employed two main strategies: direct TDP-43 silencing and indirect approaches that reduce its pathology. A study by Russo et al. (2024) developed novel polymeric nanovectors for delivering TDP-43 small interfering RNAs (siRNAs) in neuronal cells [168]. These nanovectors are typically composed of cationic polymers that bind negatively charged siRNAs via electrostatic interactions, forming more stable complexes. This condenses siRNA into particles that can efficiently penetrate cells and promote endosomal escape, thereby increasing cytoplasmic delivery of the target TDP-43. Their nanovector formulations effectively reduced TDP-43 mRNA and protein levels to levels comparable to those observed in traditional lipid-based systems [168]. This provided the first evidence that polymeric nanovectors are a viable strategy for treating TDP-43 proteinopathies by directly silencing TDP-43.

Another study revealed that Rho guanine nucleotide exchange factor (RGNEF), an RNA-binding protein, was colocalized in TDP-43 aggregates under pathological stress. Extending this observation, the authors observed that HEK293T cells transfected with RGNEF had a significant survival benefit following treatment with arsenite or sorbitol [169]. This led to the suggestion that RGNEF protects cells under pathological stress. Drawing on this evidence, the same group mapped out the interactions. It was shown that the N-terminal fragment of RGNEF (NF242) directly interacts with the RRM domains of TDP-43, and that the IPT/TIG domain of NF242 is primarily responsible for this interaction [170]. Moreover, expression of NF242 in a fruit fly ALS model that overexpressed TDP-43 mitigated the neuropathological phenotype, resulting in increased lifespan, the abolition of motor deficits, and reversal of neurodegeneration [170]. These findings revealed a protective role for RGNEF against TDP-43 under cellular stress, which could be therapeutically harnessed to regulate TDP-43 pathology in ALS.

A recent attempt to exploit the TFEB pathway through its overexpression was also made to stimulate autophagy-lysosomal activity, albeit in a GRN-knockout (GRN-KO) cell model. The authors showed that GRN-KO cells exhibited increased nuclear localization of TFEB and increased expression of lysosomal transcripts, but impaired autophagy [171]. Overexpression of TFEB increased lysosomal transcripts and partially restored autophagy. Upon injection of an adeno-associated virus (AAV) expressing mouse Tfeb into the thalamus of Grn-/-mice, lysosomal transcripts were increased, and levels of SCMAS, an indicator of lysosomal storage material, were reduced, indicating decreased lysosomal storage burden rather than loss of lysosomes [171]. Collectively, these results demonstrate that neuronal TFEB activation can pharmacologically rescue lysosomal dysfunction induced by GRN deficiency, a key upstream contributor to FTLD-TDP pathology. TFEB overexpression can likely provide mechanistic support for TDP-43 clearance strategies that exploit the autophagy-lysosomal pathway, both in GRN-related or broader TDP-43 pathologies.

Due to ongoing challenges in safely and efficiently delivering siRNAs to the CNS, one study modified endogenous small RNA processing in liver cells to generate siRNA-encapsulating small extracellular vesicles (SEVs) [172]. Initial treatment of a TDP-43 pathology (TDP-43^M337V^) mouse model using the siRNAs (IVSA-siR-TDP-43) effectively reduced TDP-43 aggregates and improved motor function and neuropathology. This was then expanded into an AAV-delivery system that contained IVSA-siR-TDP-43, resulting in sustained therapeutic effects in TDP-43-associated neurodegeneration [172]. By harnessing the patient’s own tissues as a long-term source of siRNA and leveraging endogenous RNA processing and natural vesicular trafficking across the BBB, this approach is minimally invasive, sustainable, and CNS-directed, without the delivery and safety challenges associated with traditional techniques.

### 6.4. Kinase Modulators and Signal Transduction

A growing repertoire of kinase-targeting approaches is designed to drive TDP-43 clearance by regulating upstream effectors of proteostatic pathways, rather than TDP-43 itself. IκB kinase (IKK), glycogen synthase kinase-3 beta (GSK3β), and Nemo-like kinase (NLK) are stress- and inflammation-response kinases that reconfigure PTMs, phase behavior, and trafficking, thereby influencing aberrant TDP-43 targeting by the UPS cascade or ALP [47,173,174,175]. In particular, GSK3β was documented to have dual roles in promoting autophagic clearance and suppression, depending on the pathological context [174]. For instance, under stress conditions (e.g., ischemia, energy shortage), GSK3β was shown to favor autophagic clearance of damaged proteins to promote neuronal survival [176]. Conversely, GSK3β can suppress mTOR-mediated autophagy by interacting with its upstream pathways during energy-rich or inflammatory conditions [177].

Acting in parallel are the AMPK-mTOR-TFEB, ERK, and HDAC6-linked aggresome signaling pathways, which drive autophagic flux, lysosomal biogenesis, and microtubule transport, thereby facilitating the clearance of misfolded TDP-43 and its aggregates without altering *TARDBP* expression [178,179,180,181]. These kinase- and pathway-level interventions function as intermediaries between broader proteostatic enhancers (such as mTOR and ERK) and highly selective TDP-43-directed modalities. By modulating related pathways, the proteostatic network can be redirected toward more efficient processing of aberrant TDP-43 species without directly engaging the protein itself.

## 7. Conclusions

The contributory role of TDP-43 aggregates to neurodegeneration stems either from their own toxicities due to cytoplasmic crowding or from their sequestration of biologically active proteins, rendering them functionally incapacitated. The growing literature suggests a combination of these two roles, supporting the ‘clearance deficit hypothesis,’ which posits that worsening neuropathology results not only from the overexpression of aberrant proteins but also from their failure to be cleared by the UPS and ALP systems.

While UPS mainly serves as the first line of defense by processing soluble misfolded monomers, its capacity can be easily overwhelmed in pathological states. This leads to the accumulation of ubiquitinated proteins and activates stress-response signaling, which upregulates ALP. Although the ALP’s primary role is processing larger oligomers and aggregates, it can also degrade misfolded monomers. However, it could still collapse eventually without the UPS, resulting in an uncontrolled cascade of aggregation. Most critically, a recurring theme across the studies mentioned, particularly in cellular and animal models, is a putative vicious cycle in which TDP-43 actively compromises the very endolysosomal and autophagic systems that normally clear them; in human ALS/FTD and related TDP-43 proteinopathies, convergent findings of ubiquitin-positive TDP-43 inclusions and modified autophagic markers support this association, though the exact sequence of events and causality remain unresolved. This leads to a therapeutic paradox: stimulating a compromised system still results in failed clearance if downstream ALP components and flux remain undermined.

Emerging therapeutic strategies, such as TFEB activators, PROTACs, and ASOs, are more target-specific than their broad-acting predecessors (e.g., rapamycin and rapalogs). Relying solely on activating the autophagic pathway via mTORC1 inhibition can be complicated, as mTORC1 sits at an upstream node shared with other controllers of protein synthesis (S6K, 4E-BP1), lipid and nucleotide synthesis, and cell growth [64]. Consequently, mTOR-dependent inducers may exert pleiotropic effects far beyond the scope of aggregate clearance.

**Figure 3:**
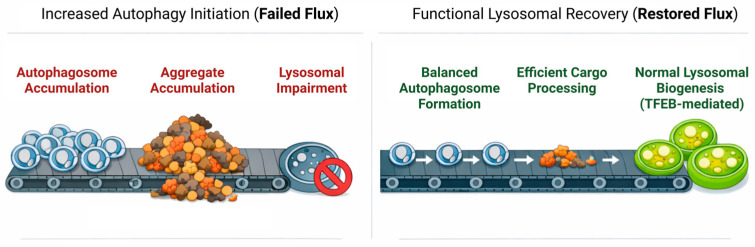
Shifting the paradigm from autophagy induction to flux restoration. Traditional therapeutic approaches often emphasize initiating autophagy, which can lead to a bottleneck of accumulated, unprocessed autophagosomes if lysosomal function is impaired. Future approaches must focus on preserving autophagic flux to ensure completion of aggregate processing. To achieve this functional recovery, it will probably be necessary to use multi-target strategies that include both metabolic stabilization and precise degradation. Created in BioRender. Angelo Jamerlan. (2025) https://BioRender.com/.

Furthermore, reports showed mixed outcomes of rapamycin treatment, with clearance of related neurodegenerative diseases demonstrated in some but failed phenotypic rescue in mouse models in others [70,71,72,73]. These inconsistent results are aligned with the vicious cycle described earlier, in which pathological TDP-43 has been proposed to sequester and functionally inactivate essential components of the lysosomal pathway, thereby limiting the impact of upstream autophagy induction. Autophagy is suppressed by mTORC1; since mTORC1 inhibition can initiate autophagy upstream, complete clearance is unlikely if autophagic flux is already compromised by the depletion or mislocalization of components (e.g., E2/E3 ligases, ubiquitin, dynactin), in which case autophagosomes accumulate without properly serving their function. In contrast, TFEB activation with small molecules specifically modulates lysosomal biogenesis, thereby ensuring that the cell catches up with TDP-43-driven sequestration by replacing the lost components (Figure 3). Even more precise approaches include ASOs and PROTACs, which directly interact with TDP-43 to regulate its expression and prevent the sequestration of elements involved in cellular clearance pathways.

The viability of prospective therapeutic strategies could be improved if these go beyond single ALP interventions. Due to the spatiotemporal heterogeneity of TDP-43 pathology, a multi-target strategy, such as administering metformin to stabilize cellular metabolism and autophagic tone while delivering more precise ASOs and PROTACs to clear aggregates, may improve current outcomes. The goal is to ensure functional recovery and preserve autophagic flux rather than relying solely on ALP upregulation, which could lead to autophagosome accumulation without degradation. This can be achieved by developing reliable biomarkers that reflect successful target engagement and lysosomal function in vivo and could be a significant step forward in the transition from bench to clinical applications. From this perspective, proximal readouts, such as the restoration of normal STMN2 splicing (loss of cryptic exon inclusion and recovery of full-length STMN2), could be reliable pharmacodynamic indicators that nuclear TDP-43 function has been restored, while more global markers like blood or CSF NfL indicate axonal injury, which are expected to decline if the incorporated clearance strategies reliably improve the disease. Combining these TDP-43-associated molecular markers with indices of lysosomal competence and flux in future trials should facilitate the improved discrimination between simple autophagy induction and complete restoration of functional flux in patients.

## Figures and Tables

**Table 1:** Summary of compounds reported to enhance autophagy, organized by strength of published evidence: Tier 3 (human trials), Tier 2 (neurodegenerative animal models), Tier 1 (in vitro studies). Arrows indicate increasing (↑) and decreasing (↓) levels.

Compound/Modality	Pharmacological Group	Primary Pharmacological Target/Pathway	Preclinical Pharmacodynamics (Model, Key Readout)	Clinical Status/Efficacy	Evidence Tier
Bosutinib	Src/c–Abl kinase inhibitor	Src/c–Abl axis, autophagy enhancement	ALS iPSC motor neurons (SOD1, TDP-43, C9orf72); ↓ misfolded SOD1, improved mitochondrial gene expression, ↑ neuron survival; SOD1 mice: modest survival benefit	Phase 1 ALS: safe/tolerated, subset with ↓ plasma NfL and slower progression; Phase II observational study ongoing	Tier 3
Ibudilast	PDE inhibitor, autophagy/ TFEB modulator	PDE inhibition of the mTORC1–TFEB axis	HEK TDP-43/SOD1 models; ↓ aggregates, ↑ TFEB nuclear translocation, ↓ mTORC1 signaling	Progressive MS: reduced brain atrophy; ALS: Phase IIb ongoing, efficacy inconclusive	Tier 3
Metformin	AMPK activator, metabolic scaffold	AMPK-dependent and -independent mTORC1 inhibition, metabolic stress	AD models (APPPS1); ↓ amyloid pathology; multiple models show autophagy modulation and mitochondrial support; no direct TDP-43 clearance data	Widely used in T2D; mixed epidemiology for AD risk; no TDP-43 clinical trials	Tier 3 (indirect)
Monepantel	mTOR-pathway modulator	Off-target inhibition of mTOR signaling, autophagy/apoptosis	Cancer and cellular models; ↑ autophagy markers	Phase 1 MEND ALS trial: attenuated ALSFRS-R decline, CSF metabolite detected, good short-term tolerability	Tier 3 (early human)
Trehalose/TFEB-targeting small molecules (trametinib, clomiphene, PKIS2 hits, NALL)	TFEB activators (MEK inhibitors, SERMs, kinase inhibitors, amino acid derivatives)	MEK–ERK–TFEB, estrogen receptor–TFEB, AKT-related kinases, TFEB stereospecific activation	5xFAD, APP/PS1, and mutant HTT models; improved cognition, ↑ autophagy–lysosome gene expression, ↑ lysosomal function, clearance of mutant HTT; NALL: rapid TFEB activation, ↑ LAMP1	Clomiphene and NALL are approved for other indications; no TDP-43-targeted trials yet	Tier 2–3 (context-dependent)
Rapamycin	mTOR-dependent autophagy inducer	mTORC1–ULK1 inhibition, broad autophagy initiation	TDP-43 transgenic mice; ↓ p62, ↑ LC3-II, improved learning/motor function, but mixed efficacy in SOD1 and AD models	No ALS disease-modifying benefit; mixed results across neurodegenerative trials	Tier 2 (cell + animal)
Tamoxifen	mTORC1 inhibitor, SER modulator	mTORC1 inhibition	TDP-43 mice; improved motor performance, changes in LC3-II and NeuN+ similar to rapamycin	No TDP-43–specific clinical data; repurposing concept only	Tier 2
Spermidine	mTOR-independent autophagy inducer	SIRT1-mediated deacetylation, global autophagy activation	Yeast, nematodes, flies; lifespan extension via autophagy; TDP-43 models: improved motor performance when combined in regimen	Nutraceutical; no dedicated ALS/FTD trials	Tier 2
Carbamazepine	Autophagy inducer	Myo-inositol depletion, PI signaling, mTOR-independent	Neuronal/macrophage models; ↑ autophagic clearance without measurable mTOR inhibition	Approved anticonvulsant; no TDP-43-specific efficacy data	Tier 2
Withaferin-A	Withanolide, NF-κB/aggresome modulator	NF-κB/NEMO autophagy-related kinases	FTLD-TDP mice (TDP-43^G348C^); ↓ RIPA-insoluble TDP-43, ↑ LC3B-II, improved cognition; inconsistent effects on Beclin-1/p62/Atg5 across studies	Herbal-derived; no ALS/FTD trials	Tier 2
IMS-088	WFA analog, NEMO antagonist	NF-κB essential modulator (NEMO), autophagy	CCH-induced vascular dementia mice with TDP-43 pathology; ↓ phospho-TDP-43 aggregates, ↑ LC3B/Beclin-1, improved cognitive and motor deficits	Preclinical only	Tier 2
Curcumin/analogs	Polyphenol, autophagy/ redox modulator	PI3K–Akt–mTOR, AMPK–SIRT1, ERK, HO-1, TFEB	TDP-43 fragment models; ↓ TDP-25 levels, ↓ oxidative stress, ↑ HO-1; TFEB-activating analog C1 drives TFEB nuclear translocation and autophagic flux; SLCP in TDP-43A315T mice ↓ phospho-TDP-43, improved survival via estrogen pathway	No TDP-43-specific trials; bioavailability and BBB penetration remains limited	Tier 2
Resveratrol	Polyphenol, SIRT1/TFEB modulator	SIRT1–TFEB (Lys116 deacetylation), ER-Ca^2+^–PP2A–TFEB	Microglial and neuronal models; ↑ TFEB nuclear translocation, ↑ lysosomal biogenesis, enhanced Aβ degradation; TDP-43 effects untested	Dietary supplement; no TDP-43 outcome data	Tier 1–2
Trehalose	mTOR-independent autophagy modulator	Calcineurin–TFEB activation, lysosomal Ca^2+^ signaling	TDP-43 cell model; ↓ TDP-43 accumulation, ↑ TFEB nuclear translocation, ↑ LC3-II; later tfLC3 studies show impaired flux and autolysosome formation	No TDP-43 clinical trials; IV required due to TREH metabolism	Tier 1–2
Trehalose analogs (melibiose, lactulose)	TFEB-dependent autophagy modulators	Calcineurin–TFEB activation, TREH-resistant	ARpolyQ and motoneuron models; ↑ TFEB nuclear translocation, ↑ clearance of polyQ proteins; TFEB knockdown abrogates the effect	Preclinical only	Tier 1–2
ASOs/siRNA platforms	Nucleic acid therapeutics	TARDBP mRNA knockdown or RNA-binding modulation	ENA-modified ASOs in ALS/FTD TDP-43 models; ↓ TDP-43 expression, improved behavior, and aggregation; siRNA SEVs/AAV platforms reduce aggregates and improve motor function in TDP-43M337V mice	Early preclinical; no approved TDP-43 ASO yet; C9orf72 antisense ASO trials show mechanism-specific nuances	Tier 1–2
EGCG	Polyphenol, anti-aggregation agent	Direct TDP-43 binding, AMPK–mTOR–ULK1 (other models)	In vitro TDP-43 binds with micromolar affinity, prevents nucleation and aggregation, favors less toxic species; autophagy modulation shown in non-TDP-43 contexts	Widely used supplement; no targeted ALS/FTD efficacy data	Tier 1
PROTACs	PROTAC	Benzothiazole-aniline binder + CRBN ligand; UPS-mediated targeted degradation	Neuro-2a C-terminal TDP-43 model; PROTAC-2 (3-EG linker) selectively degrades C-TDP-43 aggregates, rescues cytotoxicity, spares endogenous TDP-43	Preclinical; BBB penetration and E3 ligase specificity unresolved	Tier 1

**Table 2:** Practical criteria for assessing autophagic flux restoration.

Aspect	Recommended Readouts
LC3 turnover	LC3-II with ± lysosomal inhibitors (e.g., BafA1, chloroquine) to show increased LC3 degradation rather than simple accumulation.
Cargo clearance	p62/SQSTM1 levels and degradation (often with protein synthesis block) as a marker of selective cargo flux.
Vesicle maturation	Tandem fluorescent LC3 reporters (mRFP/mCherry-GFP-LC3) to distinguish autophagosomes (GFP+/RFP+) from autolysosomes (GFP-/RFP+).
Fusion/lysosome status	LC3–LAMP1/2 colocalization, lysosomal pH and cathepsin activity, LysoTracker/Magic Red assays to confirm intact fusion and lysosomal competence.
TDP-43 relevance	Parallel reduction in insoluble or phosphorylated TDP-43 species to demonstrate that enhanced flux translates into improved TDP-43 clearance.

## Data Availability

Data sharing does not apply to this article, as no new data were created or analyzed in this study, and all information is derived from previously published sources as cited.

## Abbreviations

The following abbreviations are used in this manuscript:

AAV	Adeno-associated virus
AD	Alzheimer’s disease
ALP	Autophagy–lysosome pathway
ALS	Amyotrophic lateral sclerosis
ALSFRS-R	ALS Functional Rating Scale–Revised
AMPK	AMP-activated protein kinase
APP/PS1	Amyloid precursor protein/presenilin-1 double-transgenic AD mouse model
ARpolyQ	Androgen receptor with an elongated polyglutamine tract
ASO	Antisense oligonucleotide
ATG	Autophagy-related gene/protein (e.g., ATG5, ATG9B)
BBB	Blood-brain barrier
BTA	Benzothiazole–aniline (TDP-43-binding scaffold in PROTACs)
CaMKKβ	Calcium/Calmodulin-Dependent Protein Kinase Kinase 2
CCH	Chronic cerebral hypoperfusion
CLEAR	Coordinated lysosomal expression and regulation
CMA	Chaperone-mediated autophagy
CML	Chronic myelogenous leukemia
CNS	Central nervous system
COPD	Chronic obstructive pulmonary disease
COX-2	Cyclooxygenase-2
CRBN	Cereblon
CSF	Cerebrospinal fluid
CYP19A1	Aromatase enzyme
CYP3A4	Cytochrome P450 3A4 enzyme
CYP450	Cytochrome P450 enzyme
DPR	Dipeptide repeat
DUB	Deubiquitinase
ENAs	2′-O,4′-C-ethylene nucleic acids
ERK	Extracellular signal-regulated kinase
ERK1/2	Extracellular signal-regulated kinase 1/2
ESCRT	Endosomal sorting complexes required for transport
FTD	Frontotemporal dementia
FTD-TDP	Frontotemporal dementia with TDP-43 aggregates
FTLD	Frontotemporal lobar degeneration
FTLD-U	Frontotemporal lobar degeneration with ubiquitin-positive inclusions
GAPDH	Glyceraldehyde 3-phosphate dehydrogenase
GSK3β	Glycogen synthase kinase-3 beta
HDAC6	Histone deacetylase 6
HECT	Homologous to the E6-AP carboxyl terminus
HEK	Human embryonic kidney
HO-1	Heme oxygenase-1
Hsc70	Heat shock cognate 70 kDa protein
HTT	Huntingtin protein
IKK	IκB kinase
IKKα	IκB kinase alpha
IKKβ	IκB kinase beta
IVSA	In vivo self-assembled (siRNA platform, context-specific)
KFERQ	Lys-Phe-Glu-Arg-Gln
LAMP1	Lysosome-associated membrane protein 1
Lamp2A	Lysosome-associated membrane protein 2A
LATE	Limbic-predominant age-related TDP-43 encephalopathy
LATE-NC	LATE neuropathologic change
LC3	Microtubule-associated protein 1 light chain 3
LDH	Lactate dehydrogenase
MAPK	Mitogen-activated protein kinase
MDA	Malondialdehyde
MEND	Monepantel in Motor Neurone Disease
MGAT3	Mannosyl (beta-1,4-)-glycoprotein beta-1,4-N-acetylglucosaminyltransferase
MS	Multiple sclerosis
mTOR	Mechanistic target of rapamycin
mTORC1	Mechanistic target of rapamycin complex 1
NALL	N-acetyl-L-leucine
NEDD4	Neural precursor cell expressed developmentally down-regulated protein 4
NEMO	NF-κB essential modulator
NeuN	Neuronal nuclei (RBFOX3; pan-neuronal marker)
NfL	Neurofilament light chain
NF-Κb	Nuclear factor kappa-light-chain-enhancer of activated B cells
NLK	Nemo-like kinase
NPC	Niemann–Pick disease type C
NSC-34NeuN	TDP-35 neuronal cellsNeuronal nuclei (RBFOX3; pan-neuronal marker)
PDE	Phosphodiesterase
PEG	Polyethylene glycol
PI3K	Phosphoinositide 3-kinase
PP2A	Protein phosphatase 2A
PROTAC	Proteolysis-targeting chimera
PSMC1	Proteasome 26S subunit, ATPase 1
PSMC3	Proteasome 26S subunit, ATPase 3
RAD23A	RAD23 homolog A
RGNEF	Rho guanine nucleotide exchange factor
RIPA	Radioimmunoprecipitation assay buffer
ROS	Reactive oxygen species
RRM	RNA recognition motif
RSV	Resveratrol
SCI	Spinal cord injury
SCMAS	Subunit C of mitochondrial ATP synthase
SEV	Small extracellular vesicle
SIRT1	Sirtuin 1
SLCP	Solid lipid curcumin particles
SOD1	Superoxide dismutase 1
SQSTM1	Sequestosome-1 (p62)
STMN2	Stathmin-2
TARDBP	Gene encoding TDP-43
TCP-1	T-complex protein 1
TDP-35-GFP	Green fluorescent protein tagged TDP-35 fragment
TDP-43	Transactive response DNA-binding protein of 43 kDa
TDP-43-GFP	Green fluorescent protein tagged TDP-43
TFE3	Transcription factor E3
TFEB	Transcription factor EB
TLR	Toll-like receptor
TREH	Trehalase
UBE2E	Ubiquitin-conjugating enzymes
UBPY	Ubiquitin isopeptidase Y
ULK1	Unc-51-like kinase 1
UPS	Ubiquitin–proteasome system
USP13	Ubiquitin-specific peptidase 13
VDR	Vitamin D receptor
VHL/CUL2	Von Hippel-Lindau/Cullin-2 protein complex
ZNF129	Zinc finger protein
